# The Many Faces of Elevated TSH: When to Avoid Thyroid Hormone Therapy in Older Adults

**DOI:** 10.1093/geroni/igab046.1813

**Published:** 2021-12-17

**Authors:** Jennifer Mammen, Enoch Abbey, John McGready, Luigi Ferrucci, Eleanor Simonsick

**Affiliations:** 1 Johns Hopkins University School of Medicine, Baltimore, Maryland, United States; 2 Johns Hopkins University Bloomberg School Of Public Health,, Baltimore, Maryland, United States; 3 National Institute on Aging, Baltimore, Maryland, United States; 4 National Instute on Aging/NIH, Baltimore, Maryland, United States

## Abstract

We have previously demonstrated that hypothalamic-pituitary-thyroid axis aging is characterized by several distinct patterns. An elevated thyrotropin (TSH) level (mean 5.6mIU/L) with normal free thyroxine (FT4) was present in 75 BLSA participants with at least 3 visits. Twenty-one percent had an historical pattern consistent with primary gland failure, while 13% had a pattern consistent with an HPT response to stressors (aging-adaptation). The remainder had intermediate patterns of change. FT4 >0.92pg/ml identified those in whom TSH elevations occurred with aging-adaptation with a 90.0% sensitivity and 93.8% specificity, indicating no need for therapy. In addition, among 597 participants with stable TSH levels in the reference range, being on thyroid hormone therapy increased mortality risk (IRR=1.8; 95% CI 0.9-2.1). Thus, including FT4 in the diagnostic criteria for hypothyroidism in older adults could target therapy to avoid the potential harm of reversing the aging adaptations in those who do not have true early hypothyroidism.

